# Supplementary material for: Multi‑objective optimization of nitrile rubber and thermosets modified bituminous mix using desirability approach
Source: PLoS One. 2023 Feb 21;18(2):e0281418. doi: 10.1371/journal.pone.0281418 (PMC9942971; doi:10.1371/journal.pone.0281418)
Supplement: S1 Appendix — (DOCX) [file pone.0281418.s001.docx]

**APPENDIX SECTION**

**LIST OF SUPPLEMENTARY TABLES**

Appendix Table S1: Experiment Results with input conditions

| **Samples S.NO.** | **NBR%** | **B%** | **FR%** | **ER%** | **STABILITY (kN)** | **FLOW (mm)** |
| --- | --- | --- | --- | --- | --- | --- |
| S1 | 5 | 10 | 10 | 2.5 | 13.05 | 2.63 |
| S2 | 0 | 5 | 0 | 0 | 12.3 | 2.71 |
| S3 | 10 | 10 | 5 | 2.5 | 15.63 | 3.19 |
| S4 | 10 | 0 | 0 | 0 | 12.45 | 2.54 |
| S5 | 0 | 10 | 5 | 2.5 | 14.95 | 3.05 |
| S6 | 0 | 0 | 10 | 5 | 15.35 | 3.16 |
| S7 | 0 | 10 | 5 | 0 | 14.36 | 2.93 |
| S8 | 0 | 0 | 0 | 2.5 | 12.99 | 2.89 |
| S9 | 0 | 5 | 0 | 2.5 | 12.34 | 2.64 |
| S10 | 0 | 0 | 0 | 0 | 9.91 | 2.96 |
| S11 | 0 | 5 | 0 | 0 | 13.28 | 2.71 |
| S12 | 5 | 5 | 0 | 2.5 | 13.57 | 2.77 |
| S13 | 5 | 0 | 10 | 0 | 16.02 | 3.27 |
| S14 | 10 | 0 | 10 | 0 | 15.67 | 3.31 |
| S15 | 0 | 10 | 10 | 5 | 15.48 | 3.16 |
| S16 | 0 | 5 | 5 | 0 | 13.13 | 2.68 |
| S17 | 0 | 10 | 5 | 5 | 14.95 | 3.46 |
| S18 | 5 | 5 | 0 | 5 | 15 | 3.06 |
| S19 | 10 | 0 | 10 | 5 | 15.97 | 3.26 |
| S20 | 0 | 5 | 5 | 5 | 16.25 | 3.36 |
| S21 | 5 | 10 | 5 | 0 | 15.53 | 3.17 |
| S22 | 0 | 10 | 10 | 2.5 | 13.33 | 3.13 |
| S23 | 5 | 0 | 5 | 5 | 17.44 | 3.56 |
| S24 | 0 | 0 | 10 | 2.5 | 14.36 | 2.93 |
| S25 | 10 | 10 | 0 | 0 | 13.62 | 2.78 |
| S26 | 5 | 10 | 0 | 2.5 | 14.6 | 2.98 |
| S27 | 0 | 0 | 0 | 5 | 13.88 | 2.88 |
| S28 | 0 | 0 | 10 | 0 | 13.11 | 2.77 |
| S29 | 0 | 10 | 0 | 2.5 | 14.28 | 2.71 |
| S30 | 10 | 10 | 10 | 0 | 14.88 | 3.24 |
| S31 | 10 | 10 | 0 | 5 | 15.93 | 3.25 |
| S32 | 5 | 10 | 5 | 5 | 16.95 | 3.46 |
| S33 | 5 | 5 | 5 | 5 | 16.66 | 3.59 |
| S34 | 10 | 0 | 10 | 2.5 | 15.05 | 3.04 |
| S35 | 0 | 5 | 0 | 5 | 15.4 | 3.12 |
| S36 | 5 | 0 | 10 | 5 | 15.75 | 3.28 |
| S37 | 10 | 5 | 5 | 0 | 14.87 | 3.03 |
| S38 | 5 | 5 | 0 | 0 | 12.34 | 2.62 |
| S39 | 5 | 0 | 0 | 5 | 14.21 | 2.9 |
| S40 | 10 | 5 | 0 | 0 | 13.67 | 2.79 |
| S41 | 5 | 10 | 10 | 5 | 16.4 | 3.55 |
| S42 | 10 | 0 | 5 | 0 | 16.61 | 3.39 |
| S43 | 10 | 5 | 0 | 5 | 14.5 | 2.96 |
| S44 | 0 | 10 | 0 | 0 | 12.43 | 2.62 |
| S45 | 10 | 10 | 5 | 0 | 16.76 | 3.42 |
| S46 | 5 | 5 | 5 | 2.5 | 13.52 | 2.76 |
| S47 | 10 | 0 | 5 | 5 | 16.66 | 3.4 |
| S48 | 0 | 5 | 5 | 2.5 | 13.48 | 2.75 |
| S49 | 10 | 10 | 10 | 5 | 15.68 | 3.2 |
| S50 | 5 | 0 | 10 | 2.5 | 14.6 | 2.98 |
| S51 | 10 | 5 | 0 | 2.5 | 13.72 | 2.8 |
| S52 | 5 | 0 | 5 | 0 | 14.95 | 3.05 |
| S53 | 10 | 10 | 0 | 2.5 | 15.14 | 3.09 |
| S54 | 0 | 0 | 5 | 5 | 16.84 | 3.64 |
| S55 | 0 | 10 | 0 | 5 | 16.3 | 3.53 |
| S56 | 5 | 5 | 5 | 0 | 13.92 | 2.84 |
| S57 | 0 | 10 | 10 | 0 | 12.59 | 2.57 |
| S58 | 10 | 5 | 10 | 5 | 15.73 | 3.21 |
| S59 | 0 | 5 | 10 | 2.5 | 14.16 | 2.89 |
| S60 | 0 | 0 | 5 | 0 | 12.99 | 2.81 |
| S61 | 5 | 5 | 10 | 5 | 16.12 | 3.29 |
| S62 | 10 | 10 | 5 | 5 | 17.25 | 3.77 |
| S63 | 10 | 5 | 5 | 2.5 | 14.99 | 3.06 |
| S64 | 10 | 10 | 10 | 2.5 | 14.16 | 2.89 |
| S65 | 5 | 5 | 10 | 0 | 13.46 | 2.75 |
| S66 | 0 | 0 | 5 | 2.5 | 14.26 | 2.91 |
| S67 | 5 | 0 | 0 | 2.5 | 13.72 | 2.8 |
| S68 | 5 | 10 | 5 | 2.5 | 14.9 | 3.04 |
| S69 | 10 | 0 | 0 | 2.5 | 12.81 | 2.62 |
| S70 | 10 | 5 | 10 | 0 | 13.82 | 2.82 |
| S71 | 10 | 5 | 5 | 5 | 15.58 | 3.18 |
| S72 | 10 | 5 | 5 | 5 | 15.58 | 3.18 |
| S73 | 10 | 0 | 5 | 2.5 | 16.22 | 3.31 |
| S74 | 5 | 0 | 5 | 2.5 | 16.11 | 2.88 |
| S75 | 10 | 0 | 0 | 5 | 14.5 | 2.96 |
| S76 | 5 | 0 | 0 | 0 | 12.84 | 2.62 |
| S77 | 0 | 5 | 10 | 5 | 15.73 | 3.21 |
| S78 | 5 | 5 | 10 | 2.5 | 14.6 | 2.98 |
| S79 | 5 | 10 | 10 | 0 | 15.34 | 3.13 |
| S80 | 10 | 5 | 10 | 2.5 | 12.94 | 2.64 |
| S81 | 5 | 10 | 0 | 0 | 14.95 | 3.05 |

Appendix Table S2: ANOVA for Marshall Stability

| **Source** | **Sum of Squares** | **df** | **Mean Square** | **F-value** | **p-value** | **Remarks** |  |
| --- | --- | --- | --- | --- | --- | --- | --- |
| **Model** | | 130.04 | 14 | 9.29 | 17.82 | < 0.0001 | significant |
| A-NBR | | 11.68 | 1 | 11.68 | 22.41 | < 0.0001 |  |
| B-B | | 2.03 | 1 | 2.03 | 3.89 | 0.0528 |  |
| C-FR | | 7.60 | 1 | 7.60 | 14.58 | 0.0003 |  |
| D-ER | | 45.26 | 1 | 45.26 | 86.81 | < 0.0001 |  |
| AB | | 0.0971 | 1 | 0.0971 | 0.1863 | 0.6674 |  |
| AC | | 0.1353 | 1 | 0.1353 | 0.2595 | 0.6122 |  |
| AD | | 7.36 | 1 | 7.36 | 14.12 | 0.0004 |  |
| BC | | 12.21 | 1 | 12.21 | 23.42 | < 0.0001 |  |
| BD | | 0.0360 | 1 | 0.0360 | 0.0690 | 0.7936 |  |
| CD | | 0.7631 | 1 | 0.7631 | 1.46 | 0.2306 |  |
| A² | | 2.96 | 1 | 2.96 | 5.69 | 0.0200 |  |
| B² | | 5.59 | 1 | 5.59 | 10.73 | 0.0017 |  |
| C² | | 21.92 | 1 | 21.92 | 42.04 | < 0.0001 |  |
| D² | | 8.08 | 1 | 8.08 | 15.49 | 0.0002 |  |
| **Residual** | | 34.41 | 66 | 0.5213 |  |  |  |
| Lack of Fit | | 33.93 | 64 | 0.5301 | 2.21 | 0.3622 | not significant |
| Pure Error | | 0.4802 | 2 | 0.2401 |  |  |  |
| **Cor Total** | | 164.44 | 80 |  |  |  |  |
| R²=0.79 | | std. dev. = 7.22 mean = 14.63 C.V.% =4.93 | | | | Adeq Precision=18.56 | |

Appendix Table S3: ANOVA for Flow

| **Source** | **Sum of Squares** | **df** | **Mean Square** | **F-value** | **p-value** | **Remarks** |
| --- | --- | --- | --- | --- | --- | --- |
| **Model** | 4.76 | 14 | 0.3398 | 12.35 | < 0.0001 | Significant |
| A-NBR | 0.1675 | 1 | 0.1675 | 6.09 | 0.0162 |  |
| B-B | 0.1013 | 1 | 0.1013 | 3.68 | 0.0593 |  |
| C-FR | 0.2656 | 1 | 0.2656 | 9.65 | 0.0028 |  |
| D-ER | 1.77 | 1 | 1.77 | 64.23 | < 0.0001 |  |
| AB | 0.0132 | 1 | 0.0132 | 0.4807 | 0.4905 |  |
| AC | 0.0587 | 1 | 0.0587 | 2.13 | 0.1488 |  |
| AD | 0.2079 | 1 | 0.2079 | 7.56 | 0.0077 |  |
| BC | 0.2144 | 1 | 0.2144 | 7.79 | 0.0069 |  |
| BD | 0.0718 | 1 | 0.0718 | 2.61 | 0.1109 |  |
| CD | 0.0023 | 1 | 0.0023 | 0.0820 | 0.7755 |  |
| A² | 0.0171 | 1 | 0.0171 | 0.6201 | 0.4338 |  |
| B² | 0.4193 | 1 | 0.4193 | 15.24 | 0.0002 |  |
| C² | 0.7510 | 1 | 0.7510 | 27.30 | < 0.0001 |  |
| D² | 0.6677 | 1 | 0.6677 | 24.27 | < 0.0001 |  |
| **Residual** | 1.82 | 66 | 0.0275 |  |  |  |
| Lack of Fit | 1.82 | 64 | 0.0284 |  |  | not significant |
| Pure Error | 0.0000 | 2 | 0.0000 |  |  |  |
| **Cor Total** | 6.57 | 80 |  |  |  |  |
| R²=0.72 | std. dev. = 0.17 mean = 3.0 C.V.% =5.48 | | | | Adeq.Precision=14.14 | |
