# Supplementary material for: Multi‑objective optimization of nitrile rubber and thermosets modified bituminous mix using desirability approach
Source: PLoS One. 2023 Feb 21;18(2):e0281418. doi: 10.1371/journal.pone.0281418 (PMC9942971; doi:10.1371/journal.pone.0281418)
Supplement: Attachment — Submitted filename: PONE-D-22-31489_reviewer.pdf [file pone.0281418.s002.pdf]

PLOS ONE

Multi-Objective Optimization of Nitrile Rubber and Thermosets Modified Bituminous Mix using Desirability Approach: Fabrication, and Evaluation of Marshall and Morphological characteristics

--Manuscript Draft--

**Manuscript Number:**	PONE-D-22-31489
**Article Type:**	Research Article
**Full Title:**	Multi-Objective Optimization of Nitrile Rubber and Thermosets Modified Bituminous Mix using Desirability Approach: Fabrication, and Evaluation of Marshall and Morphological characteristics
**Short Title:**	Multi-Objective Optimization of additives reinforced Bituminous Mix using Desirability Approach
**Corresponding Author:**	Elsayed Tag Eldin, phd<br>Future University in Egypt<br>Cairo, EGYPT
**Keywords:**	Nitrile Rubber; Thermosets; Modified Bitumen; Regression Analysis; SEM; EDS; Desirability Approach
**Abstract:**	A variety of materials, including waste and rubber products, have been used in road construction. The present study is focused on modified bitumen by using Nitrile rubber with different thermosets namely Bakelite, Furan Resin and Epoxy resin. The emphasis of the problem is to arrive at mix in order to maximize Marshall Stability and minimizing flow value of Modified Bituminous Concrete. The statistical investigation for regression models and Multi-Response Optimization by using the desirability approach in Design Expert Software. ANOVA analysis predicts that Nitrile Rubber, Epoxy Resin and Furan Resin are the major significant parameters for Marshall Stability and Flow Value. Multioptimization results suggested the optimal conditions are achieved at Nitrile Rubber 7.6%, Bakelite 4.8%, Furan Resin 2.5% and Epoxy Resin 2.6% for Marshall stability (MS) and Flow value (FV). SEM and EDS images of modified bitumen sample S1 (5% Nitrile Rubber+ 10% Bakelite + 10% Furan Resin + 2.5% Epoxy Resin) at magnification X50 at 100µm scale has reported that the fine surface with small pores is visible in SEM image of modified bitumen sample S1 due to the presence of Bakelite, NBR, ER and FR. Further the peak values from the EDS analysis for the modified bitumen sample S1 has exhibited the maximum value of Carbon at 91.93% and Sulphur at 4.37% rather than Nitrogen at 3.70% respectively. SEM image of modified bitumen sample S34 (10% Nitrile Rubber + 10% Furan Resin + 2.5% Epoxy Resin) with magnification X100 at 100µm scale has revealed the fine flaky shapes with small pores or large pores visible on the surface of modified bitumen sample S34 due to the absence of Bakelite and presence of nitrile, furan resin, and epoxy resin. The EDS analysis of modified bitumen sample S34 and respective peaks value has illustrated the maximum value of carbon at 88.23%, Oxygen at 6.30%, Sulphur at 2.54%, Calcium at 1.70%, and Nitrogen at 1.23% respectively. To validate the optimization results, the confirmation runs have been conducted and obtained results are within 5% error with optimal conditions.
**Order of Authors:**	Avani Chopra
	Sandeep Singh
	Abhishek Kanoungo
	Gurpreet Singh
	Naveen Kumar Gupta
	Shubham Sharma, PhD
	Sanjeev Kumar Joshi
	SAYED M. Eldin, phd
**Additional Information:**	

Powered by Editorial Manager® and ProduXion Manager® from Aries Systems Corporation

Question	Response
**Financial Disclosure**<br><br>Enter a financial disclosure statement that describes the sources of funding for the work included in this submission. Review the submission guidelines for detailed requirements. View published research articles from *PLOS ONE* for specific examples.<br><br>This statement is required for submission and **will appear in the published article** if the submission is accepted. Please make sure it is accurate.<br><br>**Unfunded studies**<br>Enter: *The author(s) received no specific funding for this work.*<br><br>**Funded studies**<br>Enter a statement with the following details:<br>• Initials of the authors who received each award<br>• Grant numbers awarded to each author<br>• The full name of each funder<br>• URL of each funder website<br>• Did the sponsors or funders play any role in the study design, data collection and analysis, decision to publish, or preparation of the manuscript?<br>• **NO** - Include this sentence at the end of your statement: *The funders had no role in study design, data collection and analysis, decision to publish, or preparation of the manuscript.*<br>• **YES** - Specify the role(s) played.<br><br>* typeset	The author(s) received no specific funding for this work.
**Competing Interests**<br><br>Use the instructions below to enter a competing interest statement for this submission. On behalf of all authors, disclose any competing interests that could be perceived to bias this work—acknowledging all financial support and any other relevant financial or non-financial competing interests.	The authors have declared that no competing interests exist.

This statement is required for submission and **will appear in the published article** if the submission is accepted. Please make sure it is accurate and that any funding sources listed in your Funding Information later in the submission form are also declared in your Financial Disclosure statement.<br><br>View published research articles from *PLOS ONE* for specific examples.<br><br>**NO authors have competing interests**<br><br>Enter: *The authors have declared that no competing interests exist.*<br><br>**Authors with competing interests**<br><br>Enter competing interest details beginning with this statement:<br><br>*I have read the journal's policy and the authors of this manuscript have the following competing interests: [insert competing interests here]*<br><br>* typeset	
**Ethics Statement**<br><br>Enter an ethics statement for this submission. This statement is required if the study involved:<br><br>• Human participants<br>• Human specimens or tissue<br>• Vertebrate animals or cephalopods<br>• Vertebrate embryos or tissues<br>• Field research<br><br>Write "N/A" if the submission does not require an ethics statement.<br><br>General guidance is provided below. Consult the submission guidelines for detailed instructions. **Make sure that all information entered here is included in the Methods section of the manuscript.**	N/A

**Format for specific study types**<br><br>**Human Subject Research (involving human participants and/or tissue)**<br>• Give the name of the institutional review board or ethics committee that approved the study<br>• Include the approval number and/or a statement indicating approval of this research<br>• Indicate the form of consent obtained (written/oral) or the reason that consent was not obtained (e.g. the data were analyzed anonymously)<br><br>**Animal Research (involving vertebrate animals, embryos or tissues)**<br>• Provide the name of the Institutional Animal Care and Use Committee (IACUC) or other relevant ethics board that reviewed the study protocol, and indicate whether they approved this research or granted a formal waiver of ethical approval<br>• Include an approval number if one was obtained<br>• If the study involved *non-human primates*, add *additional details* about animal welfare and steps taken to ameliorate suffering<br>• If anesthesia, euthanasia, or any kind of animal sacrifice is part of the study, include briefly which substances and/or methods were applied<br><br>**Field Research**<br><br>Include the following details if this study involves the collection of plant, animal, or other materials from a natural setting:<br>• Field permit number<br>• Name of the institution or relevant body that granted permission	
**Data Availability**<br><br>Authors are required to make all data underlying the findings described fully available, without restriction, and from the time of publication. PLOS allows rare exceptions to address legal and ethical concerns. See the PLOS Data Policy and FAQ for detailed information.	Yes - all data are fully available without restriction

A Data Availability Statement describing where the data can be found is required at submission. Your answers to this question constitute the Data Availability Statement and **will be published in the article**, if accepted.<br><br>**Important:** Stating 'data available on request from the author' is not sufficient. If your data are only available upon request, select 'No' for the first question and explain your exceptional situation in the text box.<br><br>Do the authors confirm that all data underlying the findings described in their manuscript are fully available without restriction?	
**Describe where the data may be found in full sentences. If you are copying our sample text, replace any instances of XXX with the appropriate details.**<br><br>• If the data are **held or will be held in a public repository**, include URLs, accession numbers or DOIs. If this information will only be available after acceptance, indicate this by ticking the box below. For example: *All XXX files are available from the XXX database (accession number(s) XXX, XXX.*).<br>• If the data are all contained **within the manuscript and/or Supporting Information files**, enter the following: *All relevant data are within the manuscript and its Supporting Information files.*<br>• If neither of these applies but you are able to provide **details of access elsewhere**, with or without limitations, please do so. For example:<br><br>*Data cannot be shared publicly because of [XXX]. Data are available from the XXX Institutional Data Access / Ethics Committee (contact via XXX) for researchers who meet the criteria for access to confidential data.*<br><br>*The data underlying the results presented in the study are available*	All relevant data are within the manuscript

from (include the name of the third party and contact information or URL).<br>• This text is appropriate if the data are owned by a third party and authors do not have permission to share the data.<br><br>* typeset	
Additional data availability information:	Tick here if the URLs/accession numbers/DOIs will be available only after acceptance of the manuscript for publication so that we can ensure their inclusion before publication.

Powered by Editorial Manager® and ProduXion Manager® from Aries Systems Corporation

Click here to access/download;Manuscript;Multi Objective bitumen.docx

Multi- Objective Optimization of Nitrile Rubber and Thermosets Modified Bituminous Mix using Desirability Approach: Fabrication, and Evaluation of Marshall and Morphological characteristics

Avani Chopra[1], Sandeep Singh[1*], Abhishek Kanoungo[2], Gurpreet Singh[3], Naveen Kumar Gupta[4], Shubham Sharma[5,6*], Sanjeev Kumar Joshi[7], Sayed M. Eldin[8*]

[1]Department of Civil Engineering, Chandigarh University, Mohali, Punjab, India. cu.avanichopra@gmail.com (AC); drsandeep1786@gmail.com (S.S.)

[2]Department of Civil Engineering, Chitkara School of Engineering & Technology, Chitkara University, Himachal Pradesh, 174103 -India. abhishek.kanoungo@chitkarauniversity.edu.in (AK)

[3]Department of Mechanical Engineering, Chandigarh University, Mohali, Punjab, India. garry.dhiman85@gmail.com (GS)

[4]Mechanical Engineering Department, Institute of engineering and technology, GLA University, Mathura-281406 (UP), naveen.gupta@gla.ac.in (NKG)

[5]Mechanical Engineering Department, University Centre for Research and Development, Chandigarh University, Mohali, Punjab, India; shubham543sharma@gmail.com, shubhamsharmacsircIri@gmail.com (SS)

[6]School of Mechanical and Automotive Engineering, Qingdao University of Technology, 266520, Qingdao, China. E-Mail: shubham543sharma@gmail.com, shubhamsharmacsircIri@gmail.com (S.S.)

[7]Uttaranchal Institute of Technology, Uttaranchal University, Dehradun 248007, India sanjeevjoshi@uttaranchaluniversity.ac.in (SKJ)

[9]Center of Research, Faculty of Engineering, Future University in Egypt, New Cairo, 11835, Egypt. elsayed.tageldin@fue.edu.eg (SME)

[*]**Correspondence:** elsayed.tageldin@fue.edu.eg (SME); drsandeep1786@gmail.com (S.S.); shubham543sharma@gmail.com, shubhamsharmacsircIri@gmail.com (S.S.)

Abstract: A variety of materials, including waste and rubber products, have been used in road construction. The present study is focused on modified bitumen by using Nitrile rubber with different thermosets namely Bakelite, Furan Resin and Epoxy resin. The emphasis of the problem is to arrive at mix in order to maximize Marshall Stability and minimizing flow value of Modified Bituminous Concrete. The statistical investigation for regression models and Multi-Response Optimization by using the desirability approach in Design Expert Software. ANOVA analysis predicts that Nitrile Rubber, Epoxy Resin and Furan Resin are the major significant parameters for Marshall Stability and Flow Value. Multioptimization results

suggested the optimal conditions are achieved at Nitrile Rubber 7.6%, Bakelite 4.8%, Furan Resin 2.5% and Epoxy Resin 2.6% for Marshall stability (MS) and Flow value (FV). SEM and EDS images of modified bitumen sample S1 (5% Nitrile Rubber+ 10% Bakelite + 10% Furan Resin + 2.5% Epoxy Resin) at magnification X50 at 100µm scale has reported that the fine surface with small pores is visible in SEM image of modified bitumen sample S1 due to the presence of Bakelite, NBR, ER and FR. Further the peak values from the EDS analysis for the modified bitumen sample S1 has exhibited the maximum value of Carbon at 91.93% and Sulphur at 4.37% rather than Nitrogen at 3.70% respectively. SEM image of modified bitumen sample S34 (10% Nitrile Rubber + 10% Furan Resin + 2.5% Epoxy Resin) with magnification X100 at 100µm scale has revealed the fine flaky shapes with small pores or large pores visible on the surface of modified bitumen sample S34 due to the absence of Bakelite and presence of nitrile, furan resin, and epoxy resin. The EDS analysis of modified bitumen sample S34 and respective peaks value has illustrated the maximum value of carbon at 88.23%, Oxygen at 6.30%, Sulphur at 2.54%, Calcium at 1.70%, and Nitrogen at 1.23% respectively. To validate the optimization results, the confirmation runs have been conducted and obtained results are within 5% error with optimal conditions.

Keywords: Nitrile Rubber, Thermosets, Modified Bitumen, Regression Analysis, SEM, EDS, Desirability Approach

Abbreviations:

DOE Design of experiments

NBR Nitrile Rubber

B Bakelite

FR Furan Resin

ER Epoxy Resin

SBR Styrene-Butadiene rubber

OBC Optimum Bitumen Content

MSMAX Maximization of Marshall Stability

FVMIN Minimization of Flow Value

ZMIN Multi-objective function minimization

G_m Bulk Specific Gravity

G_t Theoretical specific gravity

V_b Percent volume of bitumen

VFB Percent voids filled with bitumen

VMA Percent void in mixed aggregate

V_v Air Voids Percent

1. Introduction

Recent advancements in the traffic trend have opened avenues for exploring the potential for utilizing waste products to reduce the burden on consumption of bitumen for sustainable pavements. A variety of additives have been developed to improve the performance attributes of bitumen which have been used successfully in many applications [1]. To boost bitumen performance, modifiers and additives such as polymers, oxidants and antioxidants, chemical modifiers, expanders, anti-stripping additives and hydrocarbons have been used in pavements [2]. Due to addition of polymers, a chain of small molecules is repeated which results in enhanced performance of the pavement. The polymer modified bitumen increases the strength against rutting, fatigue and cracks. It also increases elastic recovery, viscosity, softening point, adhesion and flexibility [3, 4]. Moreover, modified bitumen improves the durability and breakdown due to rutting deformations in high temperature and thereby increases the fatigue life. Modified bituminous mix has become appropriate for being used as a wearing or binder course due to enrichment in the stiffness modulus, creep resistance and indirect tensile strength that are subjected to heavy traffic. Therefore, it can be effectively utilized at busy intersections, bridge decks and roundabouts to increase the life of the road surfacing.

Over the fifteen years or so the consumption of bitumen-modified roads per year has increased to almost 05 million tons, with crumb rubber-modified bitumen accounting for nearly 80% of this. The remaining 20% is consumed through the use of polymer modified bitumen. The use of Thermosetting asphaltic mixtures including bitumen modified with polymers result in increasing the adhesive as well as resistance properties, fatigue performance, and rigidity modulus [5].

Over the previous decades, researchers have made many efforts using different additives and modifiers to enhance the performance of modified bitumen in pavements. It has been found that the rubber or thermosets have been extensively used for preparing modified bituminous roads as an alternative road construction material. Chinounet. al [6] employed NBR waste (nitrile butadiene rubber)/EVA (ethylene vinyl acetate) association with 5% NBR/EVA, which led to decreased penetrability, indicating an increase in modified bitumen hardness. Al-Assi M. & Kaseem [7] done investigations on the tyre-pavement friction factor and established a clear link between the adhesive binding energy and the observed friction coefficient, indicating

that there is more low-speed friction in a rubber-pavement system. Soudani et al. [8] measured the properties of modified road bitumen with waste NBR in the sole of the shoe at high temperatures. The measurements demonstrate a satisfactory workability function and increase rheological features such as rutting resistance and shear at increased temperatures for samples having larger values of NBR waste content.

Saha & Suman S. [9] investigated the effect of bitumen60/70 grade modified with the addition of 1% to 5%Bakelite on mechanical and qualitative properties. The softening stage, complicated viscosity, and binder penetration, Marshall Stability value improved at 2% bakelite addition. Cubuk et al. [10] examined the use of phenol-formaldehyde on bitumen modification and reported that this modified mix produced increased durability against high traffic and environmental dangers due to raised flexibility as well as stripping qualities at 2% content of phenol-formaldehyde. Bostancioglu and Oruc [11] tested the Marshall properties of modified asphalt mixes made by combining activated carbon (CA) and furan resin. The results reported that addition of CA and FR substantially enhanced the Marshall's stability value by 9%, and due to addition of FR the Marshall quotient increased by 25%. In case of the stiffness modulus of indirect tensile, the mixtures containing 5% FR and 10% CA had an overall stiffness modulus of 16% higher than the conventional combination. The usage of Furan Resin content between 2% - 6% in 50/70 penetration grade bitumen enhances the ageing tolerance and consistency of bitumen and minimizes the humidity and temperature sensitivity [12].

Recently chemical additives are being used for increasing the efficacy of recycled asphalt mixtures. Bituminous mix treated with epoxy resin mixture, SBS, and PVA resulted in best pavement performance towards acid resistance resulting in fuel efficiency. The dynamic residue viscosity test revealed a potential large viscosity value for several applications of pavement [13]. Zhang et al. tested the efficiency of modified bitumen (tack coat) emulsion which was modified with aqueous ER and SBR. The inclusion of SBR and ER increased the shear strength, as well as the high and low-temperature characteristics [14]. Vyrozhemskyi et al. summarized the use of polymer additives which dramatically boost bitumen's mechanical properties which is an important element to optimize the durability of bituminous pavements vulnerable to high traffic loads [15]. The use of asphalt and bitumen modification thermosetting polymers, such as ER, is found as a possible solution and literature reveals that ER-containing asphalt pavements have improved riding capabilities. A considerable jump in the asphalt pavement strength is observed when 20-35% of bitumen is replaced with ER, especially at elevated temperatures. Ji et al. [16] investigated the quality of cold mix asphalt made with modified bitumen using waterborne epoxy. The micro-surfaces created with aqueous epoxy resin-modified bitumen emulsion also demonstrated promise resistance to moisture damage, skid, and rutting. Cubuk et al. [17] examined the rheological and efficiency attributes after introducing different quantities of epoxy in bitumen with a 50/70 penetration grade and inferred that the optimal additive dose was found to be 2% (w/w).

Various researchers utilized different tools and techniques for Optimization of the Bituminous Mixes for better pavement performance and pavement design. Gundaliya & Patel [18] utilized Genetic Algorithm to determine optimum mix for Bituminous Concrete. Tsai et al. [19] obtained satisfactory results by utilizing GA to solve non – linear optimization problems related to pavement design. Yu et al. [20] developed a model for multi-objective optimization to solve the three decision objectives maintenance arrangement of asphalt pavement such as environmental impacts, pavement performance and cost in the life cycle. Nik et al. [21] used different combinations of particle swarm optimization (PSO) and hybrid genetic algorithm (GA) to suggest an optimal solution for surveyed pavement inspection units (SIUs) to reduce cost, improve the accuracy of pavement network and minimization of inspection errors. Santos et al. [22] proposed a new Adaptive Hybrid Genetic Algorithm (AHGA) to enhance the effectiveness and overall efficiency of the search. The results revealed that the proposed AHGA statistically performed better than the traditional GA in efficiency terms. Trivedi et al. [23] developed a mechanistic-empirical rutting model prediction using genetic algorithms (GA). The model predicted that rut depths returned values with better accuracy. Sebaaly et al. [24] utilized GA to develop models for Marshall mix design for the four asphalt mix variables, i.e., flow, stability, theoretical maximum specific gravity (G_{mm}) and bulk specific gravity (G_{mb}). Liu et al. [25] did multi-response optimization using Response Surface Method (RSM) to achieve the satisfactory properties in the modified asphalt binder. Bala et al. [26] used Response Surface Methodology (RSM) for the prediction of Marshall volumetric properties of modified bitumen containing polyethylene and nanosilica. Moghaddam et al. [27] used the point prediction function in the Design-Expert software for analyzing the optimal values of polyethylene terephthalate (PET) and asphalt contents to meet the design requirements of Stone Mastic asphalt (SMA) mixtures. Hamza et al. [28] developed model for predicting the value of Optimum Binder Content (OBC) for various levels of compaction temperature and Recycled Asphalt (RA) content.

In the reported work, a combination of Nitrile Rubber and Thermosets (Bakelite, Furan Resin & Epoxy Resin) in certain proportions with bitumen for preparing the Modified Bituminous Blend has been analyzed for the evaluation of the microstructural, morphological and engineering properties of the Bituminous Mix. The main intent of this paper is to optimize the input parameter values for the Marshall stability and flow value using the desirability function approach in design expert software.

2. Materials and Methods

To prepare the modified bitumen, the various materials such as bitumen, aggregates, fillers, Nitrile Rubber, Bakelite, Furan Resin and Epoxy Resin have been used in present work. These materials along with properties are discussed in details.

2.1 Materials and their properties

2.1.1 Bitumen

Bitumen grade VG-40 was used in this research work to study the Volumetric and Marshall properties of bitumen modified with the addition of Nitrile Rubber and Thermosets (Bakelite, Furan Resin and Epoxy Resin). The physical properties of bitumen are given in Table 1.

Table 1: Physical Properties of Bitumen Grade VG-40

Property of bitumen	Test value	Range as per IS 73-2013
Penetration value (mm)	49	Minimum 35
Softening point (˚C)	53	Minimum 50
Ductility value (cm)	29	Minimum 25
Flash Point (˚C)	228	Minimum 220

2.1.2Aggregates

The crushed coarse and fine aggregates were used and several physical tests like Los Angles Abrasion test, water absorption test, aggregate impact, and crushing value tests were performed. Fig. 1 shows the gradation limits of the aggregates used in this study matching to Grade 1 of Bituminous Concrete as per MORT&H.

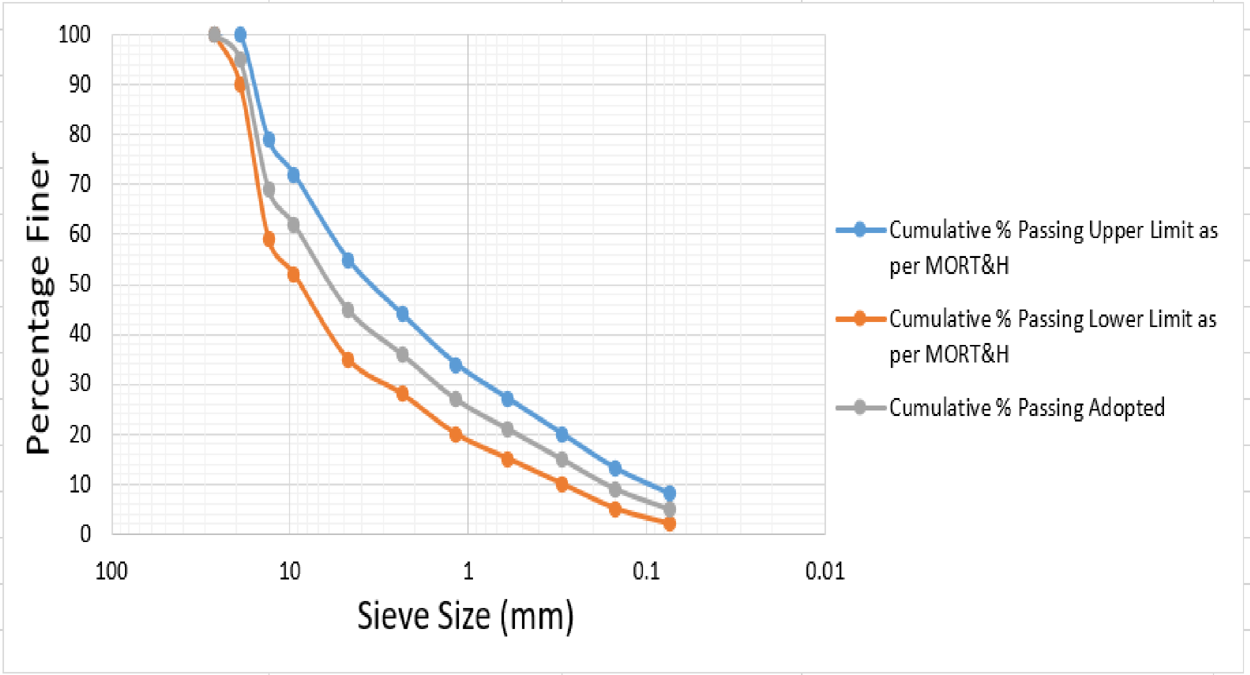

Figure 1: Gradation of aggregates used in this study

2.1.3 Filler

Stone dust has been used as the filler for preparing the conventional and modified bituminous mix. The filler should be classified within the limits set out in the Table 500-9 of MORT&H specification are mentioned in Table 2.

Table 2: Gradation of Filler

**IS Sieve (mm)**	**Cumulative Percent Passing by weight of Total Aggregate**	**Range as per MORT&H for Cumulative Percent Passing by weight of Total Aggregate**
0.6	100	100
0.3	95	95-100
0.075	90	85-100

2.1.4. Nitrile Rubber

Nitrile Rubber is the acrylonitrile butadiene rubber (NBR), obtained from the milling shoe soles an industrial waste blackish in color) [8, 30-31]. Table 3 showcases the properties of NBR as obtained from the vendor. Five characterization tests were performed on the nitrile rubber samples. The values given above are compared with the American Society of Testing and Material (ASTM) i.e., ASTM D2000, ASTM D297ASTM D573 and the test values were found to be well within the limits.

Table 3: Properties of Nitrile Rubber

**Property Name**	**Value**
Hardness	70+/-5 shore
Modulus of elasticity	Greater than or Equal to11MPa
Tensile strength	Greater than or Equal to17MPa
Density	1.28 g/cc
Temperature Range	-30 to110^{o}C

2.1.5. Bakelite

Bakelite is the trade name given to phenol-formaldehyde which is a thermosetting plastic. Table 4 shows the properties of Bakelite. As per IS 2036 – 1995 Grade P1 the tensile strength

and other tests were performed on the Bakelite sample. It has been observed that the obtained value of tensile strength, flexural strength and specific gravity were well within the laid down values as per IS 2036 -1995 Grade P1 [32-34].

Table 4: Properties of Bakelite

Property Name	Value	Values as per IS 2036
Tensile strength	1050 kg/cm^2	1050 kg/cm^2
Density	1.3 g/cc	1.3 g/cc
Flexural Strength	1350 kg/cm^2	1300 kg/cm^2 (Min)
Shear Strength	800 kg/cm^2 (750 Min)	750 kg/cm^2 (Min)
Specific Gravity	1.38	1.38

2.1.6. Furan Resin

Furan is a furfural-derived thermoset resin. Table 5 shows the properties of Furan resin sample used in this investigation. The properties were checked through experiments and then compared with the laid down corresponding limits as mentioned in IS 4832 Part II. The flexural strength, compressive strength and water absorption by weight in percentage were found to be well within the laid own limits and hence it was used in the present study [35-37].

Table 5: Properties of Furan Resin

Property Name	Value	Values as per IS 4832 Part II
Colour	Colourless	-
Flexural Strength	80 kg/cm^2	Min 75 kg/cm^2
Compressive strength	370 kg/cm^2	Min 350 kg/cm^2
Absorption by weight in percentage	0.68 %	Max 1%
Boiling point	313^oC	-

2.1.7. Epoxy Resin

Epoxy resin is a flexible usually thermosetting resin made by copolymerization of an epoxide with another compound having two hydroxyl groups and used chiefly in coatings and adhesives [29]. Table 6 shows the properties of Epoxy resin sample obtained from the supplier and it was compared with the values as laid down in IS 9197. It has been observed that the critical properties were well within the limits laid down in IS 9197 specifications.

Table 6: Properties of Epoxy Resin

Property Name	Value	Values as per IS 9197
Colour	Colourless	-
Viscosity at 27^{0}C	11 Pa s	3 to 20 Pa s
Specific gravity at 27^{0}C	1.09	0.5 to 1.20
Hydrolyzable Chorine percentage	0.40	Max 0.60
Boiling point	178°C	-

2.2 Sample preparation

The above discussed materials were used to prepare the modified bituminous mixes for the evaluation of Marshall Properties. Aggregates and filler weighing 1200 g are heated at 160-175°C. Bitumen is heated at 160-170°C and added in predetermined percentages. The mixing temperature is maintained at 160-170°C. In the pre-heated mould the mix is poured and compaction is done up to 75 blows on both the sides of the specimen by the rammer. The weight of mixed materials used in the specimen preparation can be adjusted to obtain 63.5+/-3 mm thickness. In the next trial, increase the bitumen concentration by 0.5% and retesting was done.

2.3 Determination of Optimum Bitumen Content (OBC)

In order to determine the optimum bitumen binder content, the raw bitumen VG-40 was taken for experimentation. The properties of the mix were determined based on the Marshall Mix design. The optimum bitumen content was analyzed based on the behavior of the mix by comparing it with stability, flow, air voids, VMA & VFB. In order to achieve this, the specimens were prepared to have binder content varying from 5.0 to 6.5% by weight with an incremental increase of 0.5%.

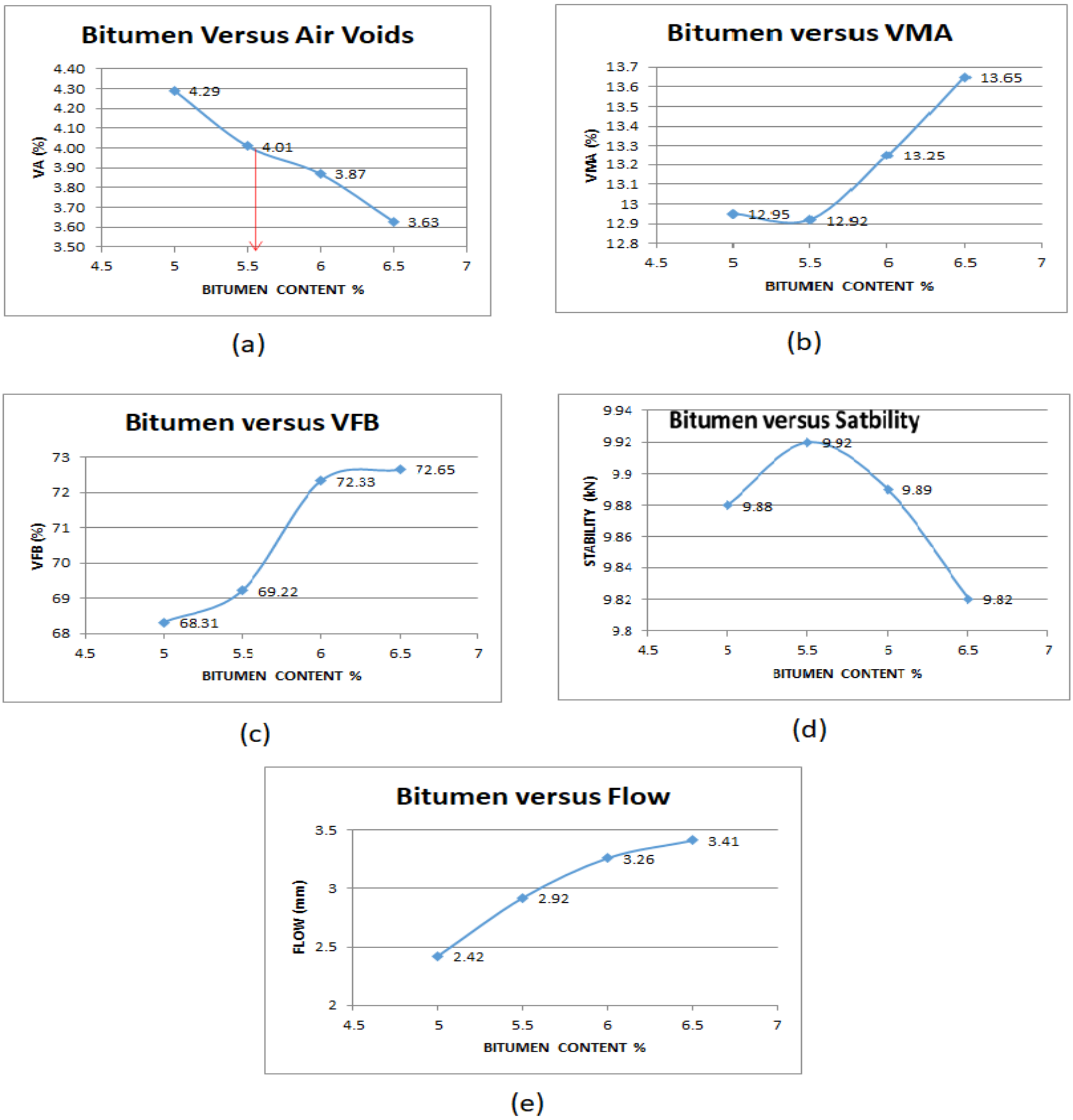

Figure 2: Volumetric Analysis for VG-40: (a) Air Voids vs Bitumen content %; (b) Voids in bitumen content (VMA) vs Bitumen content %; (c) Voids filled with bitumen (VFB) vs Bitumen content %; (d) Stability vs Bitumen content %; (e) Flow vs Bitumen content %.

The values obtained during the experimentation were noted and the same have been represented in Fig. 2. Maximum theoretical specific gravity obtained by an asphalt density tester based on ASTM D 2041 determines the maximum specific gravity of the mixture. To get the stability and flow value of specimens, Marshall Stability test was conducted based on ASTM D 6927 for BC mixtures. The recommended value for determination of OBC is corresponding to 4% Air Voids, after checking the values of the above-discussed properties to fall within the limits

of MORT&H Specifications was achieved at 5.56% of bitumen. Table 7 shows the values of the other volumetric properties which are being accepted as per the MORT&H limits.

Table 7: Volumetric Properties of Bituminous Mix at OBC of 5.56%

S.No.	Properties	Value Obtained	Permitted Values as per MORT&H Table-500-17
1	Stability (kN)	9.91	9 (minimum)
2	Flow (mm)	2.96	2 – 4
3	Marshall Quotient (MQ)	4.27	2 – 5
4	% Air Voids (VA)	4	3 – 5
5	% Voids Filled with Bitumen (VFB)	70.01	65 – 75
6	% Voids in Mineral Aggregate (VMA)	12.98	12 (minimum)
7	% Tensile Strength Ratio (TSR)	80.55	80 (minimum)

2.4 Marshall Mix Design

Marshall stability test estimates how well Marshall mix design procedure will perform at the loading rate of 50.8 mm/min, the measurement of the largest load supported by the test specimen is measured which is defined as stability. Another dial gauge measures the deformation caused by the loading which indicates the flow value.

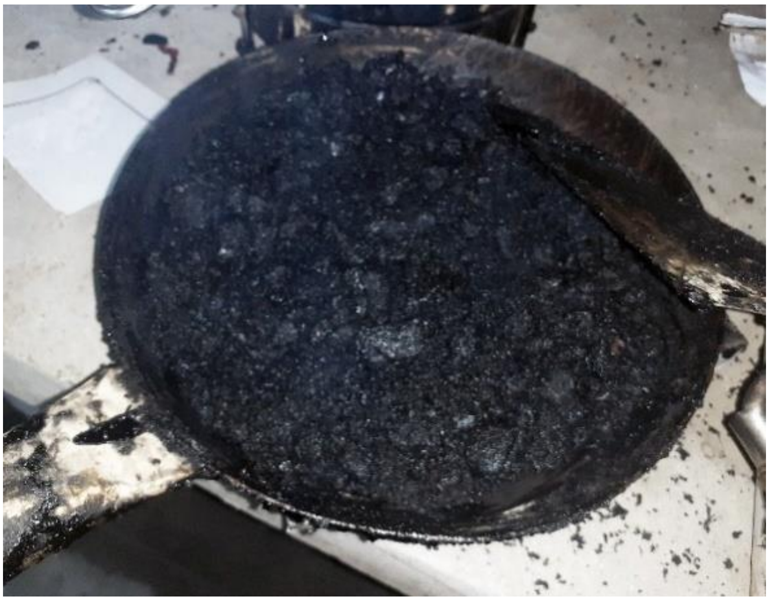

Figure 3: Mixing of aggregates, filler, modifier and bitumen

Modifiers in various combinations can be added in bitumen either by using the Dry or wet Process. In present work, the wet process was adopted to ensure homogeneous mixing and blending [38-39]. The modifiers along with bitumen were mixed by hand for 15 min and heated at 160 to 170°C before mixing with preheated aggregates (160 to 175°C). Thereafter the aggregates along with modified bitumen were transferred in a preheated mould for Marshall Testing as shown in Fig. 3.

2.5 Design of Experiment (DOE)

In this experimentation, Taguchi DOE technique is used to design the experiments. It has the advantage over traditional DOE method as it can consider multiple factors at time to determine the optimal parameters by using less experimental runs than the traditional DOE method [37-39]. To perform the investigation, the approach using the L81 orthogonal array was selected. In this method, total 243 experiments have been conducted for studying the complete space using the L81 orthogonal array.

The independent variables are NBR, B, FR and ER content, while the responses considered, are Marshall stability (MS) and flow value (FV). The preliminary experimentation and reported literature were used to select the independent variables and their range. Table8 shows the different input parameters with the selected levels for the experimentation.

Table 8: Level of Variables used during experimentation

**S.No.**	**BLEND PARAMETERS**	**LEVEL-1**	**LEVEL-2**	**LEVEL-3**
		**1**	**2**	**3**
1	NBR (%)	0	5	10
2	Bakelite (%)	0	5	10
3	Furan Resin (%)	0	5	10
4	Epoxy Resin (%)	0	2.5	5

The experiments have been conducted as per the Design of Experiment approach using Mini-tab to arrive at the Marshall Properties of the modified bituminous mix. Table 9 shows the Marshall Stability and Flow value of modified bituminous mix with various percentages of NBR and Thermosets.

Table 9: Experiment Results with input conditions

Samples S.NO.	NBR%	B%	FR%	ER%	STABILITY (kN)	FLOW (mm)
S1	5	10	10	2.5	13.05	2.63
S2	0	5	0	0	12.3	2.71
S3	10	10	5	2.5	15.63	3.19
S4	10	0	0	0	12.45	2.54
S5	0	10	5	2.5	14.95	3.05
S6	0	0	10	5	15.35	3.16
S7	0	10	5	0	14.36	2.93
S8	0	0	0	2.5	12.99	2.89
S9	0	5	0	2.5	12.34	2.64
S10	0	0	0	0	9.91	2.96
S11	0	5	0	0	13.28	2.71
S12	5	5	0	2.5	13.57	2.77
S13	5	0	10	0	16.02	3.27
S14	10	0	10	0	15.67	3.31
S15	0	10	10	5	15.48	3.16
S16	0	5	5	0	13.13	2.68
S17	0	10	5	5	14.95	3.46
S18	5	5	0	5	15	3.06
S19	10	0	10	5	15.97	3.26
S20	0	5	5	5	16.25	3.36
S21	5	10	5	0	15.53	3.17
S22	0	10	10	2.5	13.33	3.13
S23	5	0	5	5	17.44	3.56
S24	0	0	10	2.5	14.36	2.93
S25	10	10	0	0	13.62	2.78

S26	5	10	0	2.5	14.6	2.98
S27	0	0	0	5	13.88	2.88
S28	0	0	10	0	13.11	2.77
S29	0	10	0	2.5	14.28	2.71
S30	10	10	10	0	14.88	3.24
S31	10	10	0	5	15.93	3.25
S32	5	10	5	5	16.95	3.46
S33	5	5	5	5	16.66	3.59
S34	10	0	10	2.5	15.05	3.04
S35	0	5	0	5	15.4	3.12
S36	5	0	10	5	15.75	3.28
S37	10	5	5	0	14.87	3.03
S38	5	5	0	0	12.34	2.62
S39	5	0	0	5	14.21	2.9
S40	10	5	0	0	13.67	2.79
S41	5	10	10	5	16.4	3.55
S42	10	0	5	0	16.61	3.39
S43	10	5	0	5	14.5	2.96
S44	0	10	0	0	12.43	2.62
S45	10	10	5	0	16.76	3.42
S46	5	5	5	2.5	13.52	2.76
S47	10	0	5	5	16.66	3.4
S48	0	5	5	2.5	13.48	2.75
S49	10	10	10	5	15.68	3.2
S50	5	0	10	2.5	14.6	2.98
S51	10	5	0	2.5	13.72	2.8
S52	5	0	5	0	14.95	3.05
S53	10	10	0	2.5	15.14	3.09

S54	0	0	5	5	16.84	3.64
S55	0	10	0	5	16.3	3.53
S56	5	5	5	0	13.92	2.84
S57	0	10	10	0	12.59	2.57
S58	10	5	10	5	15.73	3.21
S59	0	5	10	2.5	14.16	2.89
S60	0	0	5	0	12.99	2.81
S61	5	5	10	5	16.12	3.29
S62	10	10	5	5	17.25	3.77
S63	10	5	5	2.5	14.99	3.06
S64	10	10	10	2.5	14.16	2.89
S65	5	5	10	0	13.46	2.75
S66	0	0	5	2.5	14.26	2.91
S67	5	0	0	2.5	13.72	2.8
S68	5	10	5	2.5	14.9	3.04
S69	10	0	0	2.5	12.81	2.62
S70	10	5	10	0	13.82	2.82
S71	10	5	5	5	15.58	3.18
S72	10	5	5	5	15.58	3.18
S73	10	0	5	2.5	16.22	3.31
S74	5	0	5	2.5	16.11	2.88
S75	10	0	0	5	14.5	2.96
S76	5	0	0	0	12.84	2.62
S77	0	5	10	5	15.73	3.21
S78	5	5	10	2.5	14.6	2.98
S79	5	10	10	0	15.34	3.13
S80	10	5	10	2.5	12.94	2.64
S81	5	10	0	0	14.95	3.05

3. Results and Discussion

The obtained experimental results have been analyzed using Analysis of variance (ANOVA) [38-39]. The significant quadratic models and regression equations have been developed for Marshall stability and Flow value. Table 10 represents the ANOVA results for Marshall Stability. If the p-value is less than significance level (alpha) 5%, we get to know the terms which have significant contribution on the MS and FV. The 95% confidence interval ($P<0.05$) is used to evaluate the significance of the response model and all the model terms. Terms are either linear, square or interaction. R^2 is the percentage of variation in the MS and FV value. The higher the R^2 value, the better the correlation between input and output parameters. R^2 is always between 0% and 100%. The model F-value (17.82) and p-value (<0.0001) indicate that the model is significant. It has been analyzed that NBR and ER are the major contributing parameters for Stability. Table 11 represents the ANOVA results for Flow. The model is significant with the F-value (12.35) and p-value (<0.0001). For Flow, ER and FR are the most significant parameters. R-Sq values for Stability (79%) and Flow (72%) which indicates the model fits the data very well.

Table 10: ANOVA for Marshall Stability

Source	Sum of Squares	df	Mean Square	F-value	p-value	Remarks
**Model**	130.04	14	9.29	17.82	< 0.0001	significant
A-NBR	11.68	1	11.68	22.41	< 0.0001	
B-B	2.03	1	2.03	3.89	0.0528	
C-FR	7.60	1	7.60	14.58	0.0003	
D-ER	45.26	1	45.26	86.81	< 0.0001	
AB	0.0971	1	0.0971	0.1863	0.6674	
AC	0.1353	1	0.1353	0.2595	0.6122	
AD	7.36	1	7.36	14.12	0.0004	
BC	12.21	1	12.21	23.42	< 0.0001	
BD	0.0360	1	0.0360	0.0690	0.7936	
CD	0.7631	1	0.7631	1.46	0.2306	
A^2	2.96	1	2.96	5.69	0.0200	
B^2	5.59	1	5.59	10.73	0.0017	

C^2	21.92	1	21.92	42.04	< 0.0001	
D^2	8.08	1	8.08	15.49	0.0002	
**Residual**	34.41	66	0.5213			
Lack of Fit	33.93	64	0.5301	2.21	0.3622	not significant
Pure Error	0.4802	2	0.2401			
**Cor Total**	164.44	80				
R^2=0.79	std. dev. = 7.22 mean = 14.63 C.V.% =4.93				Adeq Precision=18.56	

Table 11: ANOVA for Flow

Source	Sum of Squares	df	Mean Square	F-value	p-value	Remarks
**Model**	4.76	14	0.3398	12.35	< 0.0001	Significant
A-NBR	0.1675	1	0.1675	6.09	0.0162	
B-B	0.1013	1	0.1013	3.68	0.0593	
C-FR	0.2656	1	0.2656	9.65	0.0028	
D-ER	1.77	1	1.77	64.23	< 0.0001	
AB	0.0132	1	0.0132	0.4807	0.4905	
AC	0.0587	1	0.0587	2.13	0.1488	
AD	0.2079	1	0.2079	7.56	0.0077	
BC	0.2144	1	0.2144	7.79	0.0069	
BD	0.0718	1	0.0718	2.61	0.1109	
CD	0.0023	1	0.0023	0.0820	0.7755	
A^2	0.0171	1	0.0171	0.6201	0.4338	
B^2	0.4193	1	0.4193	15.24	0.0002	
C^2	0.7510	1	0.7510	27.30	< 0.0001	
D^2	0.6677	1	0.6677	24.27	< 0.0001	
**Residual**	1.82	66	0.0275			

Lack of Fit	1.82	64	0.0284			not significant
Pure Error	0.0000	2	0.0000			
**Cor Total**	6.57	80				
R^2=0.72	std. dev. = 0.17 mean = 3.0 C.V.% =5.48					Adeq.Precision=14.14

To analyze the interaction effects of NBR, B, FR and ER on MS and FV, 3D interaction plots were generated. Fig. 4 shows the interaction 3D plots for MS and Fig. 5 shows interaction 3D plots for flow value. Figure 4 (a) represents that the maximum stability is attained at 10% NBR and 10% Bakelite. The stability continuously increases by increasing the percentage of NBR. The main reason is that NBR is a softer material thus it has more flexibility. Also, as the B content increases beyond 5% there is a marginal dip in the Stability value as compared with the peak Stability. After 5% B level due to the combined effect of hardness and fragility the stability increases. This showed that the hardness of Bakelite and flexibility of NBR complemented to each other resulting in higher stability.

Figure 4 (b) shows that the maximum stability is attained at 10% NBR and 5% FR level. The stability continuously increases by increasing the percentage of NBR. The main reason is that NBR is a softer material thus it has more flexibility. On the other side the stability increased initially by increasing the Furan Resin content but after the FR content was increased beyond 5% the stability of the mix decreased. The main reason is that FR has a comparatively stronger bond amongst its molecules but when it is mixed with NBR the bond tends to become less adhesive and hence the stability decreases.

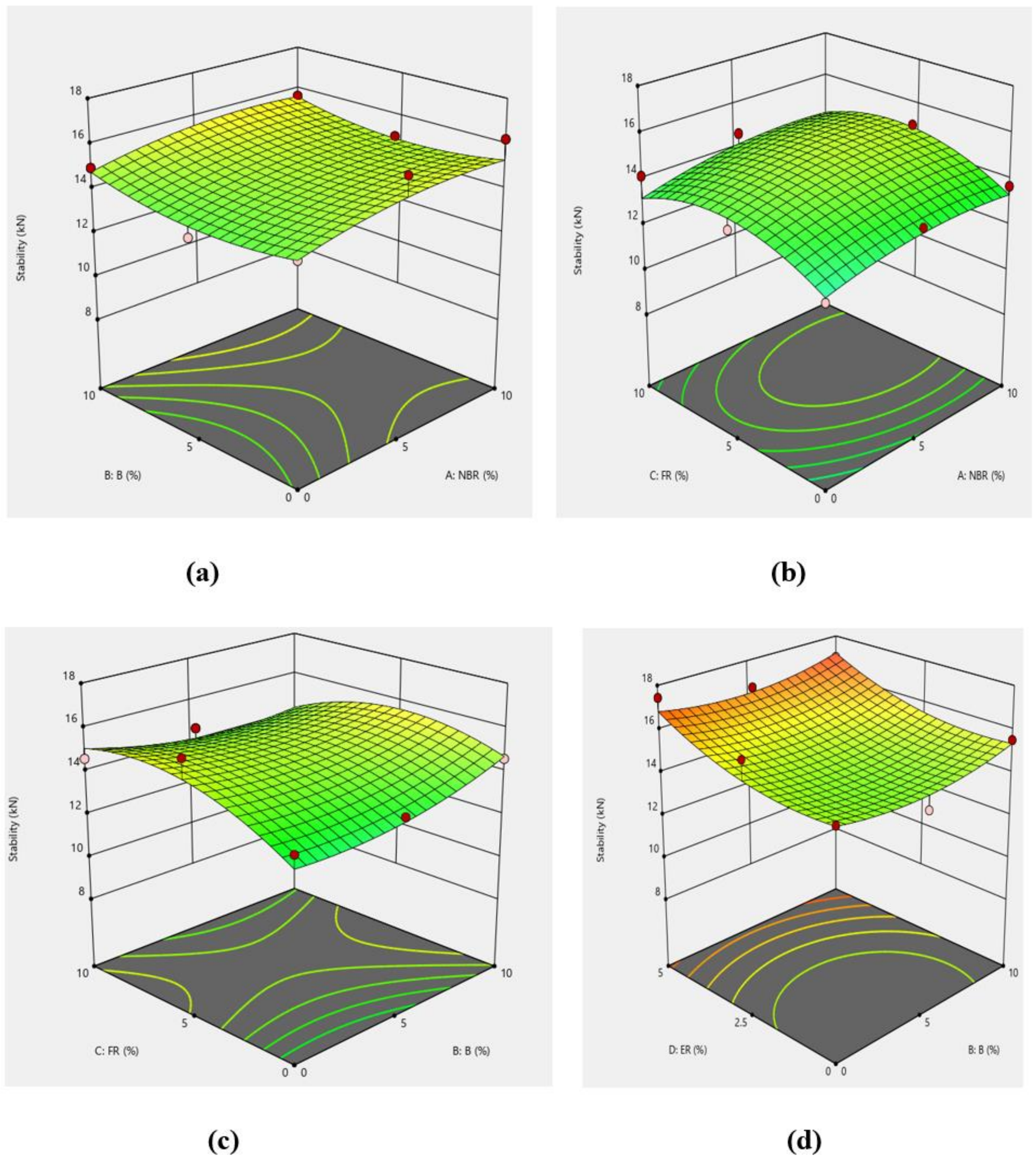

Figure 4: (a) Effect of B and NBR on Stability; (b) Effect of FR and NBR on Stability; (c) Effect of FR and B on Stability; (d) Effect of ER and B on Stability

Figure 4 (c) shows that the maximum stability is attained 10% B and 0% FR level. The stability continuously increases by increasing the percentage of FR up to the 5 % and thereafter the stability started decreasing. The main reason is that FR is having a stronger bond but with the addition of Bakelite which is a harder and brittle material it makes the mix stability value lesser. This is attributed to the fact that Bakelite is comparatively strong material and hard and brittle hence due to addition of it tends to become less adhesive and hence the stability decreases.

Figure 4 (d) shows that the maximum stability is attained at 5% ER and 10% B level. The stability continuously increases by increasing the percentage of ER and the Bakelite percentage. The main reason is that ER is having a stronger bond but with the addition of Bakelite which is a harder and brittle material, has less flexibility hence the stability decreased.

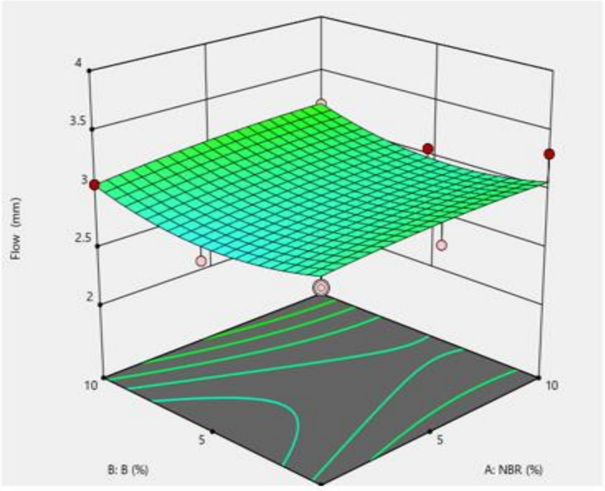

(a)

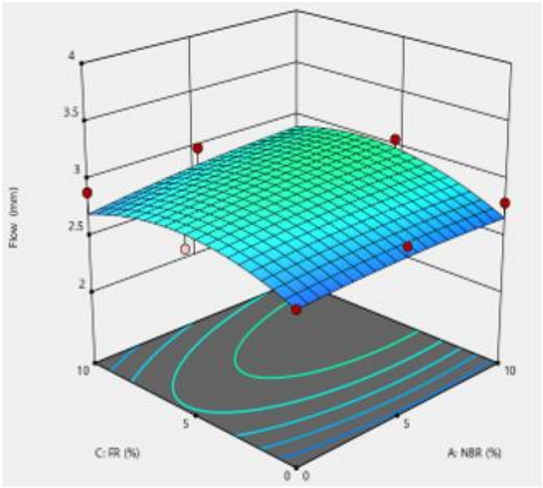

(b)

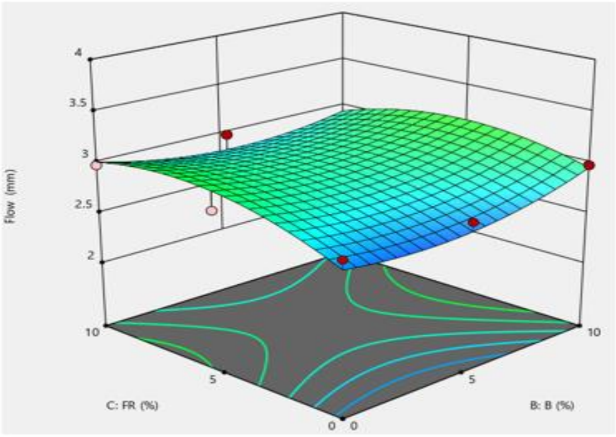

(c)

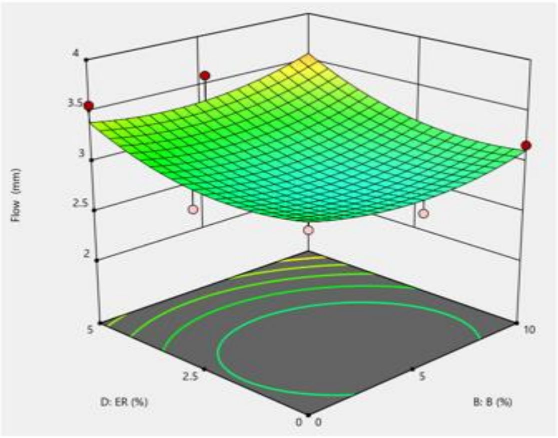

(d)

Figure 5: (a) Effect of B and NBR on Flow; (b) Effect of FR and NBR on Flow; (c) Effect of FR and B on Flow; (d) Effect of ER and B on Flow

Figure 5 (a) represents that the minimum flow is attained at 5% NBR and 5% Bakelite. The flow continuously increases by increasing the percentage of NBR. The main reason is that NBR is a softer material making the mix more flowable. Increased levels of Bakelite make the mix stiff thereby reducing the flow value.

Figure 5 (b) shows that minimum flow is obtained at 5% NBR and 0% FR level. As the NBR content is increased in the mix the flow value increase which is attributed to the flowable property associated with rubber. At equal percentages of NBR and FR that is 5% the flow is maximized. As the level of FR is further increased to 10% there is a marginal dip in the flow

value due to proper binder coating on the surface of the aggregate particles enforces stiffness in the mixture.

Figure 5 (c) represents that minimum flow is obtained at 5% B and 10% FR level. At 5% B level. On varying the FR level from 0 to 10% the flow value increases till equal levels of B and FR i.e., 5% each and thereafter the flow value again decreases marginally. The flow indicates the resistance to distortion and very high value of flow is not desirable. It is found that as the Bakelite was increased the flow started decreasing rapidly which is attributed to the fact that the stiffness of the mix is enhanced due to the addition of Bakelite.

Figure 5 (d) shows that minimum flow is obtained at 5% B and 2.5% ER level. The flow continuously increases by increasing the percentage of ER from 0 to 5%. At 2.5% ER level and on increasing the B level to 5% the flow value is reduced. The main reason is that ER is having a stronger bond but with the addition of Bakelite which is a harder and brittle material thus it has less flexibility hence the flow decreased.

4. Surface Morphology

To study the microstructure and chemical composition of modified bitumen, Scanning Electron Microscopy (SEM) has been utilized. The SEM and EDS analysis were performed on equipment Model: JEOL JSM-6610LV which having the Resolution in High vacuum Mode: 3nm, Magnification: Up to 300000, Vacuum Pressure in Chamber: Adjustable 10 - 270 Pa and Accelerating Voltage: 0.3 - 30 kV.

For SEM and EDS analysis, two best samples S1 and S34 were selected on the basis of highest Marshall Quotient (MQ) values. MQ is the ratio of Marshall Stability and Flow Value. The calculated MQ value for S1 is 4.96 and for S34 is 4.95 which were within the permitted limits as per the MORT&H specifications. MQ value specify that modified bitumen has good stiffness, resistance to creep deformation and better ability to distribute the applied load. SEM and EDS images of modified bitumen sample S1 at magnification X50 at 100µm scale is shown in Figure 6. It is clearly seen from figure 6 (a) that fine surface with small pores is visible in SEM image of modified bitumen sample S1 due to the presence of Bakelite, NBR, ER and FR. Figure 6 (b) represented the EDS of modified bitumen sample S1 and respective peaks value in the table showing the maximum value of carbon value at 91.93% and Sulphur value at 4.37% rather than Nitrogen at 3.70% respectively.

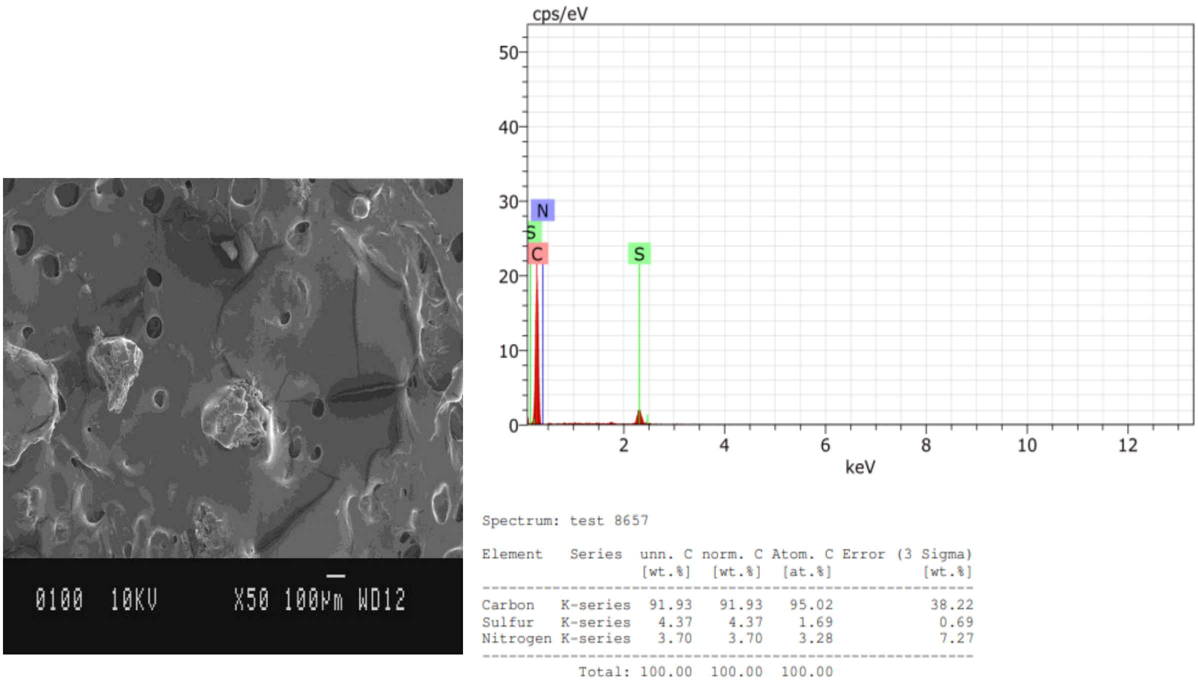

(a) (b)

Figure 6 Modified bitumen sample S1 (a) SEM image at magnification X50; (b) EDS

SEM image and EDS of modified bitumen sample S34 with magnification X100 at 100µm scale is shown in figure 7. It has been observed from figure 7 (a) that the fine flaky shapes with small pores or large pores are visible on the surface of modified bitumen sample S34 due to the absence of Bakelite and presence of nitrile, furan resin, and epoxy resin. Figure 7 (b) represented the EDS of modified bitumen sample S34 and respective peaks value in the table showing the maximum value of carbon at 88.23%, Oxygen value at 6.30%, Sulphur value at 2.54%, Calcium value at 1.70%, Nitrogen at 1.23% respectively.

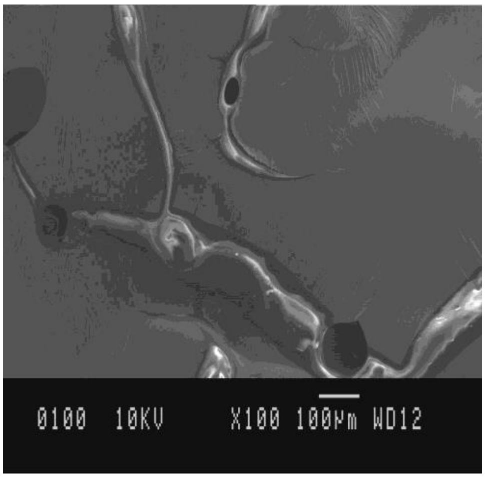

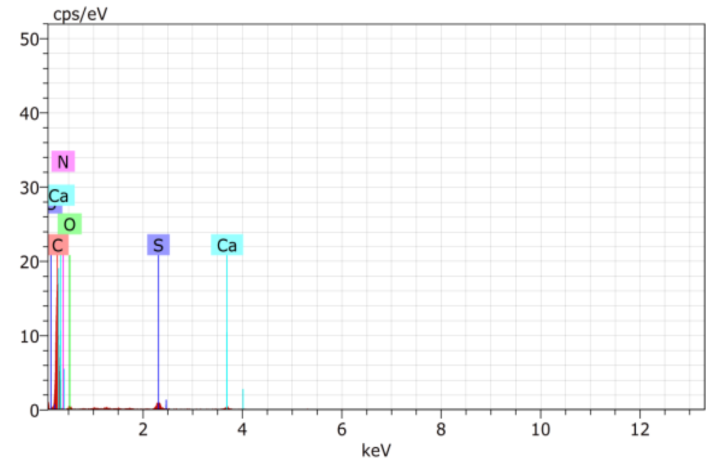

Spectrum: test 8660

Element	Series	unn. C [wt.%]	norm. C [wt.%]	Atom. C [at.%]	Error (3 Sigma) [wt.%]
Carbon	K-series	88.23	88.23	92.41	36.69
Oxygen	K-series	6.30	6.30	4.95	6.56
Sulfur	K-series	2.54	2.54	1.00	0.48
Calcium	K-series	1.70	1.70	0.53	0.41
Nitrogen	K-series	1.23	1.23	1.11	4.01
	Total:	100.00	100.00	100.00	

(a) (b)

Figure 7 Modified bitumen sample S34 (a) SEM image at magnification X100; (b) EDS

5. Multi Objective Optimization

The optimization is very essential criterion to enhance the modified mix properties [40-42]. The optimum parametric settings of MS adversely impact the FV and similarly the optimum parametric settings of FV adversely impact MS. Thus, there is a necessity to determine a unique set of parameters that provides acceptable results for the values of MS and FV. The desirability approach using design expert software has been utilized to optimize the input parameters values for MS and FV.

5.1 Regression Analysis

To carry out the regression method analysis Design Expert software was made use to investigate the impact of input factors on the output factors.

Regression equations were generated for each output responses MS and FV which are represented by equation (1), (2). The main objective is to identify a single set of Marshall variables within set limits, which would enhance the MS and minimize the FV. At first, two equations for both objective functions (MS and FV) are changed over into a single equation, as represented in (3).

$MS_{MAX} = +11.38349 + .344750NBR - 0.047712B + 0.650941FR + 0.083228ER - 0.002078(NBR)(B) + 0.002469(NBR)(FR) - 0.035880(NBR)(ER) - 0.023691(B)(FR) - 0.002573(B)(ER) - 0.011912(FR)(ER) - 0.016445(NBR)(NBR) + 0.022218(B)(B) - 0.043877(FR)(FR) + 0.107396(ER)(ER)$ ....(1)

$FV_{MIN} = +2.65864 + 0.026665NBR - 0.049292B + 0.104669FR - 0.066393ER + 0.000767(NBR)(B) + 0.001627(NBR)(FR) - 0.006030(NBR)(ER) - 0.003139(B)(FR) + 0.003635(B)(ER) - 0.000648(FR)(ER) - 0.001248(NBR)(NBR) + 0.006083(B)(B) - 0.008122(FR)(FR) + 0.030880(ER)(ER)$ ....(2)

Multi-objective Equation using equal weights:

$Z_{MIN} = -0.5 * \left(+11.38349 + .344750NBR - 0.047712B + 0.650941FR + 0.083228ER - 0.002078(NBR)(B) + 0.002469(NBR)(FR) - 0.035880(NBR)(ER) - 0.023691(B)(FR) - 0.002573(B)(ER) - 0.011912(FR)(ER) - 0.016445(NBR)(NBR) + 0.022218(B)(B) - 0.043877(FR)(FR) + 0.107396(ER)(ER)\right) + 0.5 * \left(+2.65864 + 0.026665NBR - 0.049292B + 0.104669FR - 0.066393ER + 0.000767(NBR)(B) + 0.001627(NBR)(FR) - 0.006030(NBR)(ER) - 0.003139(B)(FR) + 0.003635(B)(ER) - 0.000648(FR)(ER) - 0.001248(NBR)(NBR) + 0.006083(B)(B) - 0.008122(FR)(FR) + 0.030880(ER)(ER)\right)$ ... ...(3)

5.2 Desirability function approach

The multi-response optimization is done in two steps in desirability function approach.

i. Obtaining the desirability for the responses

ii. Amplification of desirability and identification of optimum values.

In this desirability approach, various solutions were generated by Design Expert software and solution with maximum desirability is picked. Desirability approach is used to maximize the MS and minimize the FV.

Table 12 represents the constraints utilized for process parameter optimization.

Table 12: Constraints utilized for multi response optimization

Name	Goal	Lower Limit	Upper Limit	Lower Weight	Upper Weight	Importance
A:NBR	is in range	0	10	1	1	3
B:B	is in range	0	10	1	1	3
C:FR	is in range	0	10	1	1	3
D:ER	is in range	0	5	1	1	3
Stability	maximize	9.91	17.44	1	1	3
Flow	minimize	2.54	3.77	1	1	3

Table 13 represents multi response optimization solutions to obtain maximum MS and FV. The solution with maximum desirability is selected as a best solution. As per selected solution 1, optimal input conditions are A-NBR content 7.57%, B- Bakelite content 4.79%, C-Furan Resin content 2.46% and D- Epoxy Resin content 2.58%.

Table 13: Multi response optimization solutions

Number	NBR	B	FR	D	Stability	Flow	Desirability	Remarks
1	7.573	4.793	2.463	2.581	14.482	2.879	0.663	Selected
2	7.568	4.798	2.439	2.593	14.479	2.879	0.663	
3	7.567	4.761	2.468	2.584	14.482	2.879	0.663	
4	7.521	4.810	2.453	2.579	14.480	2.879	0.663	
5	7.545	4.815	2.501	2.560	14.489	2.881	0.663	
6	5.974	2.557	10.000	2.435	14.523	2.891	0.662	
7	6.016	2.557	10.000	2.426	14.524	2.891	0.662	
8	6.004	2.549	9.998	2.442	14.529	2.892	0.662	
9	5.988	2.597	9.991	2.437	14.520	2.890	0.662	
10	5.982	2.586	9.979	2.415	14.519	2.890	0.662	

Fig. 8 represents the composite desirability of MS and FV. Design expert software was used to create the composite desirability plots. It has been analyzed from graph that the value of composite desirability is 0.66 which predicts the value of output responses for multi-response

optimization. The predicted value for MS is 14.48 kN and FV is 2.88 mm after the multi-response optimization.

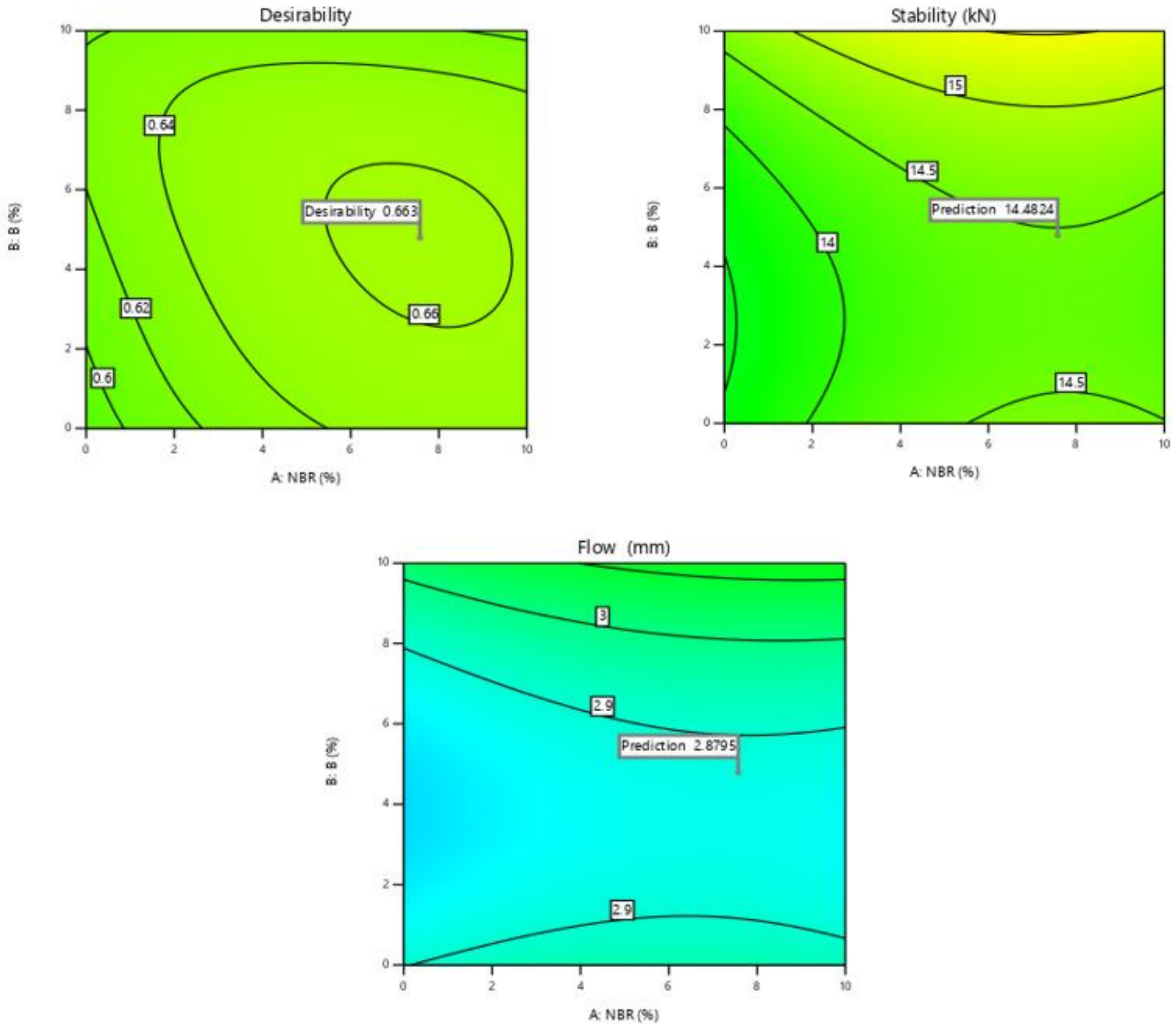

Figure 8: Composite desirability of Stability and Flow

5.3 Conformation Tests

In order to justify the optimization results the conformation test has been conducted. The conformation test has been carried out at optimum solutions to validate the characteristics for MS_{MAX} and FV_{MIN}. The results of multi-objective optimization advised to set the value of NBR 7.6%, B 4.8%, FR 2.5% and ER 2.6% to get optimum values of response parameters. The results of confirmation run for output responses are shown in Table 14.

Table 14: Response during confirmatory experiments

S.NO.	NBR (%)	B (%)	FR (%)	ER (%)	MS (kN)			FV (mm)		
					Exp	Pred	% Error	Exp	Pred	% Error
1	7.573	4.793	2.463	2.581	14.84	14.48	2	2.93	2.88	2
2	7.573	4.793	2.463	2.581	14.7	14.48	2	2.79	2.88	3
3	7.573	4.793	2.463	2.581	14.92	14.48	3	2.83	2.88	2
4	7.573	4.793	2.463	2.581	14.89	14.48	3	2.81	2.88	2
5	7.573	4.793	2.463	2.581	14.7	14.48	2	2.84	2.88	1

After the confirmation test, it was found that the average values for MS and FV are 14.81 kN and 2.84 mm respectively, which also shows that the average percentage error is less than 5% which is a desirable characteristic.

6. Conclusions

The modified bituminous mix has been successfully prepared by using four different modifiers Nitrile Rubber and Thermosets. The desirability approach has been implemented to determine optimum conditions for getting desired results. The points mentioned below are summarized from present research work:

i. The regression model shows a good correlation with the experimental results. The NBR, ER and FR are the major significant parameters for MS and FV.

ii. Multi Objective optimization using desirability approach suggests that the optimal conditions are obtained at NBR 7.6%, B 4.8%, FR 2.5% and ER 2.6% for MS and FV.

iii. SEM and EDS images of modified bitumen sample S1 (5% Nitrile Rubber+ 10% Bakelite + 10% Furan Resin + 2.5% Epoxy Resin) at magnification X50 at 100µm scale has reported that the fine surface with small pores is visible in SEM image of modified bitumen sample S1 due to the presence of Bakelite, NBR, ER and FR. Further the peak values from the EDS analysis for the modified bitumen sample S1 has exhibited the maximum value of Carbon at 91.93% and Sulphur at 4.37% rather than Nitrogen at 3.70% respectively.

iv. SEM image of modified bitumen sample S34 (10% Nitrile Rubber + 10% Furan Resin + 2.5% Epoxy Resin) with magnification X100 at 100µm scale has revealed the fine flaky shapes with small pores or large pores visible on the surface of modified bitumen sample S34 due to the absence of Bakelite and presence of nitrile, furan resin, and epoxy resin. The EDS analysis of modified bitumen sample S34 and respective peaks value has illustrated the maximum value of carbon at 88.23%, Oxygen at 6.30%, Sulphur at 2.54%, Calcium at 1.70%, and Nitrogen at 1.23% respectively.

v. Confirmation test conducted as per solution confirmed that the results for all output responses are within 5% error with optimal conditions.

Author Contributions: Conceptualization, AC, Sandeep Singh (SS), AK, GS, NKG, Shubham Sharma (SS); methodology, AC, Sandeep Singh (SS), AK, GS, NKG, Shubham Sharma (SS); formal analysis, AC, Sandeep Singh (SS), AK, GS, NKG, Shubham Sharma (SS); investigation, AC, Sandeep Singh (SS), AK, GS, NKG, Shubham Sharma (SS); writing—original draft preparation, AC, Sandeep Singh (SS), AK, GS, NKG, Shubham Sharma (SS); writing—review and editing, Shubham Sharma (SS), SKJ, SME; supervision, Shubham Sharma (SS), SKJ, SME; project administration, Shubham Sharma (SS), SME; funding acquisition, Shubham Sharma (SS), SME. All authors have read and agreed to the published version of the manuscript.

Acknowledgments: The authors would like to acknowledge the support of Future University in Egypt for paying the Article Processing Charges (APC) of this publication.

Institutional Review Board Statement: Not applicable.

Informed Consent Statement: Not applicable.

Data Availability Statement: No data were used to support this study.

Conflicts of Interest: The authors declare no conflict of interest.

References

[1] Memon, A. M., Sutanto, M. H., Napiah, M., Khan, M. I., &Rafiq, W. (2020). Modeling and optimization of mixing conditions for petroleum sludge modified bitumen using response surface methodology. *Construction and Building Materials, 264,* 120701.

[2] Porto, Michele, Paolino Caputo, Valeria Loise, Shahin Eskandarsefat, BagdatTeltayev, and Cesare Oliviero Rossi. "Bitumen and bitumen modification: A review on latest advances." *Applied Sciences* 9, no. 4 (2019): 742.

[3] Yildirim Y. Polymer modified asphalt binders. Construction and Building Materials. 2007;21(1):66-72

[4] Becker, Y.; Méndez, M.P.; Rodríguez, Y. Polymer Modified Asphalt. Vis. Tecnol. 2001, 9, 39–50.

[5] Modified bitumen with thermosetting polymers resins - Big Chemical Encyclopedia (chempedia.info)

[6] Chinoun, M., Soudani, K., & Haddadi, S. (2022). Physical and Rheological characterization of modified bitumen by NBR/EVA association. Innovative Infrastructure Solutions, 7(1), 1-8.

[7] Al-Assi M. and Kaseem E. (2017),' Evaluation of Adhesion and Hysteresis Friction of Rubber Pavement System', Journal of Applied Science, Vol: 7, 1-13.

[8] Soudani K., Cerezo V. and Haddadi S. (2016), 'Rheological characterization of bitumen modified with waste nitrile rubber (NBR)', Journal of Construction and Building Materials, Vol: 104, 126-133.

[9] Saha S. K., and Suman S. K. (2017), 'Characterization of bakelite-modified bitumen', Innovative Infrastructure Solutions, Vol: 2(01), 3.

[10] Cubuk M., Guru M., Cubuk K. M. and Arslan D. (2014),' Rheological properties and performance evaluation of phenol formaldehyde', Journal of Materials in Civil Engineering, Vol: 26(06), 1-6

[11] Bostancioglu M. and Oruc S. (2015). 'Effect of activated carbon and furan resin on asphalt mixture performance', Road materials and Pavement Design, Vol: 17 (02), 512 – 525.

[12] Bostancioğlu M. and Oruc S. (2015),'Effect of furfural-derived thermoset furan resin on the high-temperature performance of bitumen', Journal of Road Materials and Pavement Design, Vol: 16(1), 227-237.

[13] Abd El-Rahman A.M.M., El-Shafie M., Abo-Shanab Z.L., El-Kholy S.A. Modifying asphalt emulsion with different types of polymers for surface treatment applications. Pet. Sci. Technol. 2017; 35:1473–1480. Doi: 10.1080/10916466.2017.1344709.

[14] Zhang Q., Xu Y., Wen Z. Influence of water-borne epoxy resin content on performance of waterborne epoxy resin compound SBR modified emulsified asphalt for tack coat. Constr. Build. Mater. 2017; 153:774–782. Doi: 10.1016/j.conbuildmat.2017.07.148.

[15] Vyrozhemskyi V., Kopynets I., Kischynskyi S. and Bidnenko N. (2017), 'Epoxy asphalt concrete is a perspective material for the construction of roads', IOP Conference Series: Materials Science and Engineering, Vol: 236, 1-6.

[16] Ji J., Liu L., Suo Z., Xu Y., Yang S., Xu S. Performances of micro-surfacing with waterborne epoxy resin modified emulsified asphalt. J. Chang'an Univ. (Nat. Sci. Ed.) 2017; 37:8.

[17] Cubuk, M., Gürü, M., & Çubuk, M. K. (2009). Improvement of bitumen performance with epoxy resin. Fuel, 88(7), 1324-1328.

[18] Gundaliya, P., & Patel, A. Mix design optimization of bituminous concrete using genetic algorithm. Highway research, 9.

[19] Tsai, B. W., Kannekanti, V. N., & Harvey, J. T. (2004). Application of genetic algorithm in asphalt pavement design. *Transportation research record*, *1891*(1), 112-120.

[20] Yu, B., Gu, X., Ni, F., &Guo, R. (2015). Multi-objective optimization for asphalt pavement maintenance plans at project level: Integrating performance, cost and environment. *Transportation Research Part D: Transport and Environment*, *41*, 64-74.

[21] Nik, A. A., Nejad, F. M., & Zakeri, H. (2016). Hybrid PSO and GA approach for optimizing surveyed asphalt pavement inspection units in massive network. *Automation in Construction*, *71*, 325-345.

[22] Santos, J., Ferreira, A., & Flintsch, G. (2019). An adaptive hybrid genetic algorithm for pavement management. *International Journal of Pavement Engineering*, *20*(3), 266-286.

[23] Trivedi, J., Kumar, R., & Iyyunni, C. Rutting Prediction of Flexible Pavement Using Genetic Algorithm.*3rdConference of Transportation Research Group of India.*

[24] Sebaaly, Haissam, Sudhir Varma, and James W. Maina. "Optimizing asphalt mix design process using artificial neural network and genetic algorithm." *Construction and Building Materials* 168 (2018): 660-670.

[25] Liu, H., Zhang, M., Jiao, Y., & Fu, L. (2018). Preparation parameter analysis and optimization of sustainable asphalt binder modified by waste rubber and diatomite. *Advances in Materials Science and Engineering*, *2018*.

[26] Bala, Nura, MadzlanNapiah, and Ibrahim Kamaruddin. "Nanosilica composite asphalt mixtures performance-based design and optimisation using response surface methodology." *International Journal of Pavement Engineering* 21, no. 1 (2020): 29-40.

[27] Moghaddam, T. B., Soltani, M., Karim, M. R., & Baaj, H. (2015). Optimization of asphalt and modifier contents for polyethylene terephthalate modified asphalt mixtures using response surface methodology. *Measurement*, *74*, 159-169.

[28] Hamzah, M. O., Gungat, L., & Golchin, B. (2017). Estimation of optimum binder content of recycled asphalt incorporating a wax warm additive using response surface method. *International Journal of Pavement Engineering*, *18*(8), 682-692.

[29] Merriam-Webster. (n.d.). Epoxy resin. In *Merriam-Webster.com dictionary*. Retrieved January 24, 2022, from https://www.merriam-webster.com/dictionary/epoxy%20resin.

[30] Tiwary, A.K.; Singh, S.; Kumar, R.; Chohan, J.S.; Sharma, S.; Singh, J.; Li, C.; Ilyas, R.A.; Asyraf, M.R.M.; Malik, M.A. Effects of Elevated Temperature on the Residual Behavior of Concrete Containing Marble Dust and Foundry Sand. *Materials* **2022**, *15*, 3632. https://doi.org/10.3390/ma15103632

[31] Qiu, Y.; Wang, Z.; Pan, J.; Hu, F.; Sharma, S.; Deifalla, A.F. Optimal Design of Semirigid Connection Steel Frame with Steel Plate Shear Walls Using Dolphin Echolocation Algorithm. *Buildings* **2022**, *12*, 1735. https://doi.org/10.3390/buildings12101735

[32] Singh, G.; Tiwary, A.K.; Singh, S.; Kumar, R.; Chohan, J.S.; Sharma, S.; Li, C.; Sharma, P.; Deifalla, A.F. Incorporation of Silica Fumes and Waste Glass Powder on Concrete Properties Containing Crumb Rubber as a Partial Replacement of Fine Aggregates. *Sustainability* **2022**, *14*, 14453. https://doi.org/10.3390/su142114453

[33] Sharma, T.; Singh, S.; Sharma, S.; Sharma, P.; Gehlot, A.; Shukla, A.K.; Eldin, S.M. The Use of Marble Dust, Bagasse Ash, and Paddy Straw to Improve the Water Absorption and Linear Shrinkage of Unfired Soil Block for Structure Applications. *Materials* **2022**, *15*, 7786. https://doi.org/10.3390/ma15217786

[34] Rajawat, A.S.; Singh, S.; Gangil, B.; Ranakoti, L.; Sharma, S.; Asyraf, M.R.M.; Razman, M.R. Effect of Marble Dust on the Mechanical, Morphological, and Wear Performance of Basalt Fibre-Reinforced Epoxy Composites for Structural Applications. *Polymers* **2022**, *14*, 1325. https://doi.org/10.3390/polym14071325

[35] Tiwary, A.K.; Singh, S.; Kumar, R.; Sharma, K.; Chohan, J.S.; Sharma, S.; Singh, J.; Kumar, J.; Deifalla, A.F. Comparative Study on the Behavior of Reinforced Concrete Beam Retrofitted with CFRP Strengthening Techniques. *Polymers* **2022**, *14*, 4024. https://doi.org/10.3390/polym14194024

[36] Sharma, T.; Singh, S.; Sharma, S.; Sharma, A.; Shukla, A.K.; Li, C.; Zhang, Y.; Eldin, E.M.T. Studies on the Utilization of Marble Dust, Bagasse Ash, and Paddy Straw Wastes to Improve the Mechanical Characteristics of Unfired Soil Blocks. *Sustainability* **2022**, *14*, 14522. https://doi.org/10.3390/su142114522

[37] Singh, S.; Khairandish, M.I.; Razahi, M.M.; Kumar, R.; Chohan, J.S.; Tiwary, A.; Sharma, S.; Li, C.; Ilyas, R.A.; Asyraf, M.R.M.; Zakaria, S.Z.S. Preference Index of Sustainable Natural Fibers in Stone Matrix Asphalt Mixture Using Waste Marble. *Materials* **2022**, *15*, 2729. https://doi.org/10.3390/ma15082729

[38] Paul, S.; Chattopadhyaya, S.; Raina, A.K.; Sharma, S.; Li, C.; Zhang, Y.; Kumar, A.; Tag-Eldin, E. A Review on the Impact of High-Temperature Treatment on the Physico-Mechanical,

Dynamic, and Thermal Properties of Granite. *Sustainability* **2022**, *14*, 14839. https://doi.org/10.3390/su142214839

[39] Kumar, M.; Anand, A.; Chatterjee, R.; Sharma, S.; Maiti, T.K.; Dwivedi, S.P.; Saxena, A.; Li, C.; Eldin, E.M.T. Investigation on Carbonation and Permeability of Concrete with Rice Hush Ash and Shop Solution Addition. *Materials* **2022**, *15*, 6149. https://doi.org/10.3390/ma15176149.

[40] Aditya Kumar Tiwary, Sakshi Bhatia, Sandeep Singh, Jasgurpreet Singh Chohan, Raman Kumar, Shubham Sharma, S. Chattopadhyaya, S. Rajkumar. Performance comparison and critical Finite element based experimental analysis of various forms of reinforcement retaining structural system. Mathematical Problems in Engineering. 2022. Volume 2022, Article ID 4434679, https://doi.org/10.1155/2022/4434679.

[41] Manish Kumar, Jitu Kujur, Rajeshwari Chatterjee, Somnath Chattopadhyaya, Shubham Sharma, Shashi Prakash Dwivedi, Ambuj Saxena, S. Rajkumar, Ashutosh Anand. Corrosion zones of rebar in high volume flyash concrete through potentiodynamic study in concrete powder solution extracts: A sustainable construction approach. Advances in Civil Engineering. 2022. Volume 2022. Article ID 5927819. https://doi.org/10.1155/2022/5927819.

[42] Branesh Robert, Angeline Prabhavathy, Joanna P.S, Christopher Ezhil Singh, Sivaraj Murugan, Rajkumar S, Shubham Sharma. Flexural Behaviour of RC Beams with Circular Opening at the Flexural Zone and Shear Zone strengthened using Steel Plates. Advances in Civil Engineering, 2021, ID 6733402. Volume 2021, Article ID 6733402, https://doi.org/10.1155/2021/6733402
